# Aneurism

**Published:** 1828-04

**Authors:** 


### ANEURISM.

### *Femoral and Popliteal Aneurism. External Iliac Artery tied.*

William Smith, aged forty-two, admitted into the Nottingham General Hospital, August 24th, 1824, under the care of Mr. W. Wright, with an aneurism of the femoral artery, at the upper part of the thigh, and another in the ham, both in the left extremity. He gives the following history of them:—About a year ago, after having taken a walk of some miles, he felt himself much fatigued, having been in a bad state of health for some time previously, but imagined that rest would soon restore him: in two or three days after this he felt some pain in the thigh, and afterwards a small lump arose, on applying his hand to which he perceived a violent pulsation. In a short time the other tumor made its appearance in the ham, being attended with the same beating feel as the first. He says both the tumors had been gradually increasing, attended with occasional pain, till about six weeks ago, when the one in the thigh became very painful, and began to increase more rapidly, at the same time the one in the ham began gradually to decrease: from this time he could never distinguish any pulsation in either tumor.

His health appears greatly impaired; he is much emaciated, and cannot rest on account of the pain in the thigh. The aneurism has now obtained a great size, extending from about two inches below Poupart's ligament to nearly half way down the thigh: in its greatest circumference it measures twenty-one and a half inches, while that of the opposite thigh is only thirteen inches. The tumor is extremely hard, and the integuments covering it very tense, and marked with several dark purple spots: no pulsation can be felt in any part of it, but, with the assistance of the stethoscope, a sound, like water forced through a contracted channel, may be heard synchronous with the pulse at the wrist; the pulsation of the artery above the tumor is distinct, but weak. The tumor in the leg is about the size of a goose's egg: no pulsation or sound can be discovered in it by the stethoscope; it is free from pain.

Cold applications directed to be constantly used.

After this time he appeared gradually to improve: his health became better, the pain in a great measure subsided, and the sound heard by the stethoscope entirely disappeared. He took occasional doses of castor-oil, and an opiate at night when restless. The tumor evidently diminished in size, the integuments less tense, and the tumor in the ham became not more than half the size it was. In this state he remained till the 17th of August, when, attempting to raise himself up in bed, all the former symptoms returned with increased violence: the tumor became larger and more painful than ever; the sound of the pulsation was again heard, but in a few hours afterwards it disappeared, and the integuments became much more discoloured. The state of the patient becoming worse, a consultation was held on the 19th, when it was determined that the external iliac artery should be tied.

On the 22d, at twelve o'clock, Mr. Wright proceeded to operate according to Mr. C. Bell's method, by making an incision about four inches long, and nearly in a direction from the internal abdominal ring to the superior anterior spinous process of the lium: three arteries were divided and tied, two being small and superficial, the other nearly in the situation of the epigastric.

The peritoneum being now separated and held back, the external iliac was distinctly seen pulsating, and two large veins running obliquely over it; one of which was ruptured while separating the artery from its attachments. The sheath of the vessel was now opened, and one strong silk ligament passed round the artery and ruptured vein, and both were tied together. The lips of the wound were brought together by one suture in the centre and adhesive plaster. The patient was then carried to bed, and the thigh well supported by pillows under the knee.

He took twenty-four drops of the Black Drop (equal to about 100 of the Tinct. Opii); and was directed to keep quiet, and have the legs covered with flannel.

He gradually recovered; and was made an out-patient on the 18th of January.

April 12th, the following is the report:—The tumor has considerably diminished; he can walk about, but is very weak, and complains of pain in the stomach and of general dyspeptic symptoms. The wound is completely cicatrised, but there is a little descent of intestine through the opening made in the tendon of the external oblique: he attributes the descent of it to a severe cough which he has had some time. Is advised to come into the hospital, on account of his general health.

From this time to the 15th of April, he remained nearly in the same state, complaining of dyspnœa and palpitation, with cough and expectoration. He became gradually more emaciated, and died suddenly on the morning of the 15th. The body was examined the following day.

The tumors in the thigh and ham were diminished in size, and were of a firm and elastic feel; the tumor in the thigh was covered with a firm compact fascia, which was, however, but little adherent to the tumor; the posterior part of the tumor was firmly attached to the bone and neighbouring muscles by strong ligamentous substance; the external iliac was pervious from its origin to the ligature, which had completely obliterated the canal; the knot and loop of the ligature were found by the side of the artery enveloped in a little cyst resembling an inguinal gland; below the ligature, the artery was pervious to its entrance into the tumor. The tumor contained some dark-coloured, rather fluid stuff, resembling coffee-grounds; the parietes of the tumor were firm, hard, and ligamentous, and lined with layers of coagulated lymph, of the same colour as the semifluid contents; the tumor was almost filled up at the posterior part. About the entrance and exit of the artery, it was firm and cartilaginous: the artery presented nothing remarkable between the tumor in the thigh and that in the ham, which was about the size of a pullet's egg, and contained the same kind of matter as the other; its parietes were much thinner; the division of the artery below the ham was as usual. On examining the chest, the left side was found filled with coagulated blood, which had escaped from a small opening in the walls of a large aneurism of the arch of the aorta; the left lung was condensed, and the larger bronchia filled with pus; the right lung was perfectly healthy.

The above account is condensed from that published in the last Number of the *Medical Repository*.

---

*Case of Inguinal Aneurism, in which the external Iliac Artery was tied.*

Patrick Connell, æt. thirty-eight, a tailor, was admitted into St. George's Hospital, February 15th, 1828, under the care of Mr. Brodie. He stated that, in the month of October last, he was much exposed to cold and damp in Whitecross Prison, and at that time was affected with "rheumatic pains," particularly in the left leg. About the middle of November, whilst making some exertion, he felt something "give way" in the left groin, and soon afterwards noticed a small pulsating tumor there. It increased gradually in size for the first fortnight, and then remained stationary, or nearly so, until the beginning of the present month, when it became much more swollen and painful, and the limb generally œdematous. He applied eight leeches to the groin, with some relief, and took a purge or two; but has done nothing further for the complaint, and has followed his ordinary occupation until very lately.

Such was the history of the disease given by the patient; and the following were the symptoms upon his admission:—In the left groin was a hard pulsating tumor, nearly filling up the triangular space between the sartorius and pectinæus muscles. The margins of the tumor was tolerably well defined; its form was triangular, and its surface irregular, being more prominent above and below than in the centre. It appeared to be somewhat abruptly bounded by Poupart's ligament above; and extended downwards for better than two inches, probably to the point where the profunda femoris is given off. It was reducible in a great measure by pressure; the pulsation was distinct, and apparently very near the surface, showing that no great quantity of coagulum had been deposited. Pressure upon the external iliac artery completely arrested the pulsation of the tumor, but did not materially diminish its size; whilst pressure on the femoral below only lessened the pulsation. The whole limb was greatly swollen and rather tense, while the foot was œdematous and numb. There was much stiffness, with tingling, and pain shooting from the groin round to the outer side of the thigh, and down to the outside of the knee. His health had always been good; but he looked sallow and anxious, had been suffering many privations and a great deal of mental distress. The appetite was indifferent; he did not sleep well; the bowels were costive, tongue red, and the pulse had the aneurismal jerk.

Under these circumstances, he was bled once or twice, and purged; and on the 21st, the pulse being quieter, and the patient anxious for the operation, the external iliac artery was tied by Mr. Brodie.

The method of operating was that which has been recommended by Mr. Abernethy: the incision, however, being somewhat semilunar, and placed rather more on the iliac side of the vessel than in that gentleman's operations. The oblique and transversalis muscles were carefully cut through, the peritoneum cautiously raised from the belly of the iliac and psoas muscles, and the artery discovered pulsating on the inner side of the latter. Care was required in passing the needle round the vessel, in consequence of its having contracted some adhesions to the vein, &c.; but this being done, and the ligature, which was a single one, drawn tight, all pulsation ceased immediately in the tumor, though it did not diminish much in size. The lips of the wound were brought together by three sutures and adhesive straps; one end of the ligature brought out at the wound, the other having been cut short; and the patient removed to bed. The operation was performed with great facility, and the patient bore it remarkably well; whilst the pain in the thigh, &c. almost instantaneously ceased. In the evening the limb was colder than the other; he had been sick, and was restless. Pulse a little harder than in the morning. A flannel roller had been applied.

22d.—Has passed a better night, but his appearance is far from being satisfactory this morning: his breathing is oppressed; he speaks in an under tone, as if fearful of calling into action the respiratory muscles; and there is that playing of the nostrils described by Mr. Charles Bell as marking an insidious affection of the chest. There is pain in the right side and loins on taking in a full breath, as well as in attempting to cough, which he is afraid to do; thirst; tongue coated; pulse ninety, corded, and full. Mr. Brodie, on seeing the patient at one P.M., immediately directed a vein to be opened, and, after eighteen ounces of blood had been abstracted, the pain in the side was relieved, and the countenance clearer.

**H. Sennæ, sextis horis.**

Vespere.—The bleeding has had a most decided effect, the pain being much relieved, and the hardness of the pulse diminished. He has been asleep during a great part of the day, and feels much better this evening. The bowels have not yet been opened. The blood drawn is buffed.

**Repet[r] Haust. Sennæ.**

From this time he went on favorably.

In a clinical lecture delivered upon this case, immediately after the performance of the operation, Mr. Brodie observed, that he had preferred Mr. Abernethy's operation in this instance, because he was induced to believe that the disease had extended up beneath Poupart's ligament, requiring the ligature to be applied from the upper portion of the external iliac. In Sir ASTLEY COOPER's method, the ligature is necessarily applied rather low, and there is likewise a danger of wounding the epigastric artery, which was done in 1821 by M. DUPUYTREN. For these, and other reasons which we need not particularise here, Mr. B. was induced to prefer, in this instance, Mr. Abernethy's operation, which he had twice previously performed with success. He had made the incision somewhat semilunar, partly because such a form of incision affords facilities to the operator, and partly because the iliac vessels assume a certain curve as they wind round the cup of the ilium.

